# Deformation pathway and defect generation in crystals: a combined group theory and graph theory description

**DOI:** 10.1107/S2052252518017050

**Published:** 2019-01-01

**Authors:** Yipeng Gao, Yunzhi Wang, Yongfeng Zhang

**Affiliations:** a Department of Fuel Modeling and Simulation, Idaho National Laboratory, 2525 Fremont Avenue, Idaho Falls, Idaho 83415, USA; b Department of Materials Science and Engineering, Ohio State University, 2041 College Road, Columbus, Ohio 43210, USA

**Keywords:** crystal deformation, crystalline defects, group theory, Cayley graph

## Abstract

The generation and motion of crystalline defects during plastic deformation are critical processes that determine the mechanical properties of a crystal. The types of defect generated are not only related to the symmetry of a crystal but also associated with the symmetry-breaking process during deformation. Proposed here is a new mathematical framework to capture the intrinsic coupling between crystal symmetry and deformation-induced symmetry breaking. Using a combination of group theory and graph theory, a general approach is demonstrated for the systematic determination of the types of crystalline defect induced by plastic deformation, through the construction of a crystal deformation group and a deformation pathway graph. The types of defect generated in the deformation of a face-centered cubic crystal are analyzed through the deformation pathway graph and compared with experimental observations.

## Abstract 2

This work provides a new theoretical approach based on group theory and graph theory to capture the intrinsic properties of crystalline materials upon deformation.

## 1. Introduction   

The mechanical properties of a crystal, such as its strength, ductility, toughness *etc.*, are dictated by the generation and motion of defects during plastic deformation (Read, 1953 ▸; Christian & Mahajan, 1995 ▸; Kaplan, 2015 ▸; Anderson *et al.*, 2017 ▸). It has been well understood that the types of defect generated (such as dislocations and twins) are associated with the symmetry of the crystals (Bhagavantam & Suryanarayana, 1949 ▸; Nye, 1985 ▸; Prince, 2004 ▸; Muller, 2013 ▸; Gao *et al.*, 2017 ▸). As a rigorous mathematical tool to describe crystal symmetry, group theory has been widely utilized to analyze the type and crystallographic equivalency of defects (Cahn, 1977 ▸; Dmitriev & Toledano, 1996 ▸; Cayron, 2007 ▸, 2016 ▸; Gao *et al.*, 2016 ▸, 2017 ▸). Note that crystalline defects could be related to either point symmetry or translational symmetry (or a combination of the two). For example, a twin is usually associated with a point symmetry, while a dislocation is associated with a translational symmetry. In this sense, the type of defect can be interpreted as an intrinsic attribute of a crystalline state.

The generation and motion of crystalline defects represent certain displacement fields, via which plastic deformation (deformation for short hereafter) involves symmetry breaking (Ericksen, 1980 ▸; Serra *et al.*, 1988 ▸; Bhattacharya *et al.*, 2004 ▸). In other words, the displacement induced by the generation and/or motion of the defects responsible for the deformation is not only related to the symmetry of the crystalline states before and after the deformation, but also determined by the symmetry-breaking process during deformation. A typical example is the so-called lattice-invariant deformation (LID) (Bowles & Wayman, 1972 ▸; Olson & Cohen, 1979 ▸), which is a symmetry-breaking process but does not change the crystal lattice. Mathematically, an LID can be treated as a mapping relation that maps a crystal lattice onto itself through a deformation. In this regard, an LID is similar to a crystal symmetry operation that maps a crystal lattice onto itself through point and translational symmetry operations. Thus, LID is clearly a subject of group theory description. In fact, a combination of LID and crystal symmetry leads to a new group that will be referred to as a crystal deformation group (CDG) hereafter. The CDG depends on the choice of the LID, which cannot be captured in either the point group or the space group of a crystal. For a given crystal, the energetically most favorable deformation mode (associated with a specific symmetry-breaking LID process usually obtained by energetic calculations) is a piece of information beyond the geometry of a crystal, which should be a critical input for the crystallographic analysis of deformation. In other words, a CDG is constructed to reflect the coupling between the crystal symmetry and the most favorable deformation mode of a crystal. From a physical point of view, symmetry breaking is represented by a space (sometimes called order parameter space), the structure of which dictates the types of defect generated during the symmetry-breaking process. For example, the dimensionality of topological defects is determined by the topology of the order parameter space (Mermin, 1979 ▸). In general, the structure of a CDG is difficult to determine. We propose a new mathematical description based on graph theory. By applying a graph homomorphism on the Cayley graph (Cayley, 1878 ▸) of a CDG, we construct the so-called deformation pathway graph (DPG), which can be conveniently used to predict defect structures during crystal deformation.

In this paper, we establish a theoretical foundation to describe the symmetry breaking associated with LID using a combination of group theory and graph theory. Through the construction of a CDG and a DPG, we formulate a systematic approach to identify possible defect structures generated during deformation. Using a face-centered cubic (f.c.c.) crystal as an example, we demonstrate a series of Σ twin boundaries, which are compared with experimental observations. Thus, the DPG provides a new theoretical tool for tailoring material properties through defect engineering.

## 2. Construction of crystal deformation group and deformation pathway graph   

To illustrate the idea at an intuitive level, we will first show (i) how to construct a CDG (*D*) using group theory and (ii) how to construct a DPG (*G^H^*) using graph theory, for a generic 2D example. Theoretically, a CDG is the coupling result of the crystal symmetry operations and an LID operation of a crystal. To visualize the group structure of the CDG, we construct its Cayley graph (Cayley, 1878 ▸). The Cayley graph includes all the information of the CDG, some of which could be redundant for predicting defect structures. Thus, we further simplify the Cayley graph to a DPG. By combining the CDG, the DPG and the compatibility condition, deformation-induced defect structures can be systematically predicted, which is especially convenient for engineering purposes (with no requirement for knowledge of group theory). As a typical example of our new approach, systematic analysis of the CDG and DPG of an f.c.c. crystal is performed, followed by a summary of the general procedure to develop CDGs and DPGs.

Here, we first consider a square crystal lattice in 2D. The point group of a square lattice is 4*mm*, which can be represented by 2 × 2 matrices as follows:
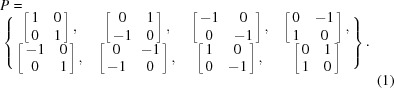
The operation (group law) of the group is matrix multiplication, and all the elements in *P* are unitary matrices with determinant 1 or −1. Each element in *P* corresponds to a symmetry operation that maps the square lattice onto itself. If we want to focus on proper rotations, only matrices with determinant 1 are considered, which gives a new group,
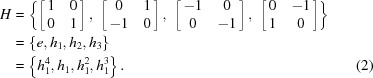
Clearly, *H* is a cyclic group of order 4, which can be generated by *h*_1_ or *h*_3_. In the following, *H* is used instead of *P* because all the deformation matrices should have a positive determinant of 1 (volume unchanged). Furthermore, the internal structure of *H* is not the focus of our paper, as will be discussed below.

**Figure 1:**
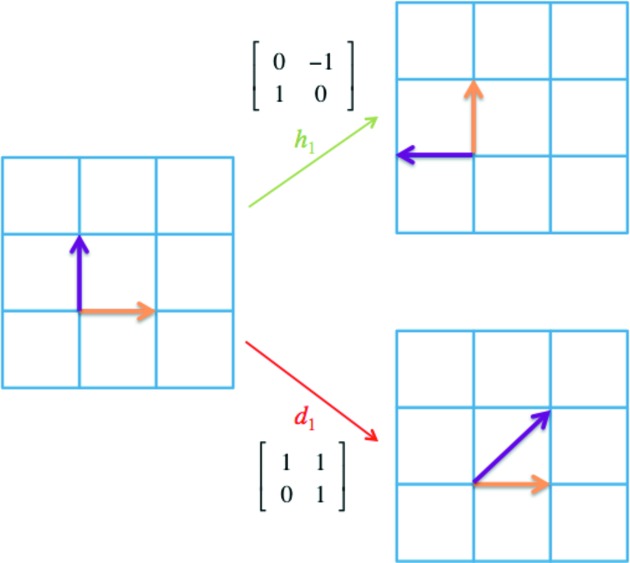
A geometric illustration of the crystal symmetry operation (*h*_1_) and the lattice-invariant deformation operation (*d*_1_) of a square lattice in 2D.

In addition, we consider an LID operation described by the following matrix,
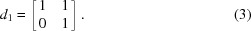
Similar to the elements in *H*, *d*_1_ also corresponds to an operation that maps the square lattice onto itself. However, such a symmetry operation is associated with the translational symmetry of the lattice. Crystallographically equivalent deformations of *d*_1_ can be determined by the combination of *d*_1_ and *H*,
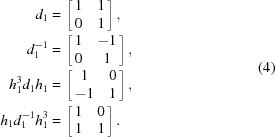
Note that all the above LIDs map a square lattice onto itself with the same orientation before and after deformation, which is critical for constructing a CDG. The geometric meanings of *h*_1_ and *d*_1_ are shown in Fig. 1 ▸. A set of two linear independent vectors in 2D (colored orange and purple) is chosen. Both crystal lattices before and after the operation (*h*_1_ or *d*_1_) are the square lattice. However, considering the change in lattice vectors before and after the operation, *h*_1_ corresponds to a 90° counter-clockwise rotation, while *d*_1_ corresponds to a shear deformation with a [10] Burgers vector on the (01) shear plane. Note that *h*_1_ and *d*_1_ are independent, since *h*_1_ is a rigid-body rotation while *d*_1_ is a shear deformation of the square lattice.

The above symmetry operations (including rotation and LID) do not change the orientation of the crystal lattice. Therefore, they can be applied to the square lattice repeatedly, which leads to the construction of a crystal deformation group *D*. It is clear that such a group is generated by *h*_1_ (the generator of the proper rotation group *H*) and *d*_1_ (the LID operation). In fact, it can be proved that *D* is exactly the SL_2_(Z) group in this example. Here, SL_2_(Z) is called the special linear group in 2D, which is represented by 2 × 2 matrices with all entries being integers (Z) and the determinant being 1 (‘special’). The group SL_2_(Z), lying discretely on SL_2_(R), has a role similar to that of Z on R (Z is an integer and R a real number). SL_2_(Z) is generated by *h*_1_ and *d*_1_ through matrix row/column operations, the proof of which can be found in textbooks and papers on group theory (Alperin, 1993 ▸; Kassel & Turaev, 2008 ▸; Rankin, 1977 ▸; Schenkman, 1965 ▸).

**Figure 2:**
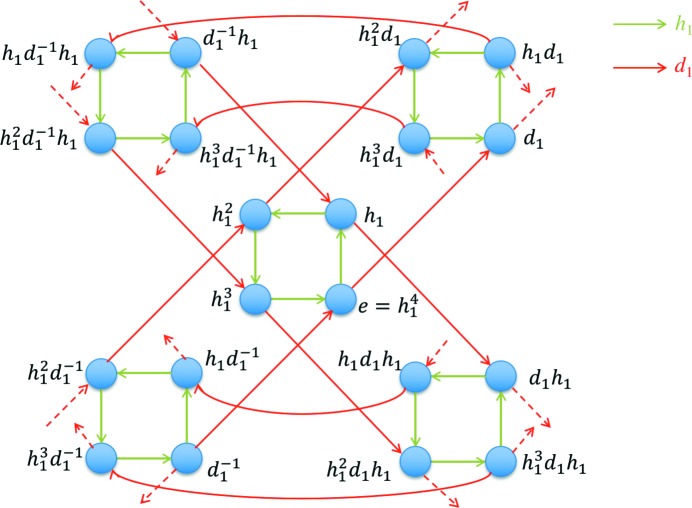
A part of the Cayley graph *G* of the crystal deformation group *D* with a specified set of generators {*h*_1_, *d*_1_}.

The structure of the group *D* (generated by *h*_1_ and *d*_1_) can be represented by its Cayley graph *G*. A Cayley graph (also known as a Cayley color graph) is a colored directed graph that captures the abstract structure of a group (Cayley, 1878 ▸). Each vertex in the Cayley graph is associated with a group element, and two vertices are connected by an edge if there exists a group generator that links the two corresponding group elements. The generation procedure of the Cayley graph of group *D* is presented as follows. The generators of *h*_1_ and *d*_1_ are represented by the directed edges in green and red, respectively, in Fig. 2 ▸. Each vertex in *G* corresponds to an element of group *D*. Since *D* is an infinite group, *G* has to be an infinite graph, so only a part of *G* is shown in Fig. 2 ▸. Here we can see individual ‘green squares’ connected by red edges. The green square captures the crystal symmetry with no consideration of deformation, which conveys the local information of graph *G* (as well as group *D*). The connections by the red edges capture the global connectivity among the green squares, which conveys the non-local information of graph *G* (as well as group *D*).

From Fig. 2 ▸, a few relations about *h*_1_ and *d*_1_ can be easily figured out, as dictated by the coupling between the crystal symmetry and the LID. For example,

(i) *h*_1_^2^ *d*_1_ = *d*_1_ *h*_1_^2^;

(ii) (*h*_1_ *d*_1_)^3^ = (*d*_1_ *h*_1_)^3^ = *e*;

(iii) (*h*_1_ *d*_1_^−1^)^3^ = (*d*_1_^−1^ *h*_1_)^3^ = *h*_1_^2^.

From a geometric point of view, all four vertices within the same green square in Fig. 2 ▸ represent the same structural state, since rigid-body rotations do not change a deformation state (analogous to objectivity or frame invariance in continuum mechanics). If the local information from the crystal symmetry (*e.g.* the green square) is neglected, we can focus on the deformation of the crystal. Here we consider a partition of the crystal deformation group *D*. It is clear that the rotation group *H* is a subgroup of *D*. Consider an element *d*_*i*_ of *D*, and the right coset of *H* in *D* is *Hd*_*i*_. All these cosets partition the entire group *D* into equal-sized non-overlapping sets.
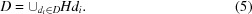
It can be easily checked that *H* is not a normal subgroup of *D*, and the right cosets {*Hd*_*i*_:*d*_*i*_ ∈ *D*} (*i.e.* *D*/*H*) do not form a group.

**Figure 3:**
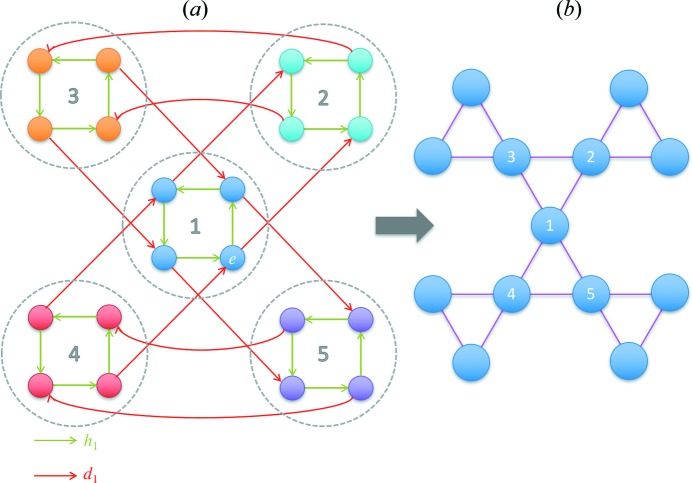
A schematic illustration of the graph homomorphism *G* → *G^H^*. (*a*) *G* and (*b*) *G^H^*.

To visualize the internal structure of *D*/*H*, we consider a graph homomorphism *G* → *G^H^* (Hahn & Tardif, 1997 ▸). Here we color the vertices in *G* with a criterion: the two vertices directly connected by *h*_1_ (green edges) are in the same color [Fig. 3 ▸(*a*)]. In addition, the vertices in the same color (circled by gray dashed lines) in *G* become a new vertex in *G^H^* and all the green edges in *G* are removed, while the red edges in *G* become undirected and unparalleled. In other words, between any two given vertices in *G^H^*, there is at most one undirected edge connecting them. The deformation pathway graph *G^H^* is shown in Fig. 3 ▸(*b*). From a group theory point of view, *G* includes all the information of group *D*, while *G^H^* captures the structure of *D*/*H* (by neglecting the internal structure of *H*). For the convenience of further analysis, we number the vertices in *G^H^. G^H^* is an infinite interconnected graph. Note that each edge in *G^H^* could correspond to one or several operations of the form *h*_1_^*m*^ *d*_1_ *h*_1_^*n*^ (*m* and *n* are integers between 0 and 3 since the cyclic order of *H* is 4 in the case of Fig. 3 ▸), *e.g.* the undirected pink edge connecting vertices 1 and 2 in *G^H^* corresponds to the two directed red edges of *e* → *d*_1_ and *h*_1_^2^ → *h*_1_^2^ *d*_1_ in Fig. 2 ▸. The triangular circuit formed by vertices 1, 2 and 3 in *G^H^* is dictated by the relation (*h*_1_ *d*_1_)^3^ = (*d*_1_ *h*_1_)^3^ = *e*, which is an intrinsic property of the deformation in a square lattice.

With the deformation pathway graph *G^H^*, we can determine the types of crystalline defect that may possibly be generated during deformation by introducing the geometric compatibility condition. In Fig. 3 ▸(*b*), each vertex corresponds to a unique structural state, and the relation between two structural states determines the possible types of defect. For convenience, we define any two vertices directly connected by an edge as the first-nearest-neighbor (1st-NN) vertices and any two vertices connected through another vertex (also through two edges) as the 2nd-NN vertices, and so on. In Fig. 3 ▸(*b*), for example, vertices 1 and 2 are 1st-NNs and vertices 2 and 4 are 2nd-NNs. Theoretically, the number of NNs indicates the number of repeated activations of the deformation mode *d*_1_. Considering two domains (in two structural states) with a planar boundary, we can determine the types of defect (*e.g.* dislocation or twin boundary). For the boundary between two domains in the structural states represented by vertices 1 and 2 (1st-NNs) in Fig. 3 ▸(*b*), the defects can be determined through the kinematic compatibility condition (Wechsler *et al.*, 1953 ▸; Bowles & Mackenzie, 1954 ▸; Wayman, 1964 ▸; Bhattacharya, 2004 ▸),
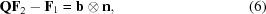
where **F**_1_ and **F**_2_ are the deformation gradient matrices of structural states 1 and 2 [corresponding to vertices 1 and 2 in Fig. 3 ▸(*b*)], respectively. In this case, **F**_1_ = *e* (identity) and **F**_2_ = *d*_1_, which are the inputs. **b** and **n** are the shear vector and shear-plane normal to be determined, respectively. **Q** is a rigid-body rotation to be determined in the solution, and 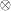 is the dyadic product operator. Note that the choices of **F**_1_ and **F**_2_ are not unique. For example, **F**_1_ can also be assigned as *h*_1_, *h*_2_ or *h*_3_. Similarly, **F**_2_ can also be assigned as *h*_1_ *d*_1_, *h*_2_ *d*_1_ or *h*_3_ *d*_1_. It can easily be proved that the choices of **F**_1_ and **F**_2_ do not affect the solutions of **Q**, **b** and **n**, in terms of crystallographic equivalency.

**Figure 4:**
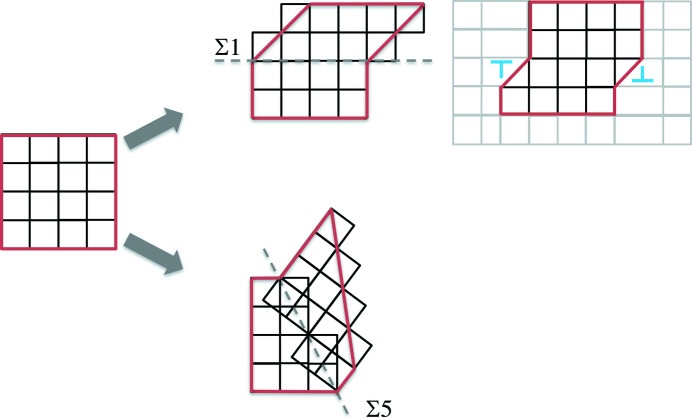
The formation of twin boundaries and dislocations between vertices 1 and 2 of Fig. 3 ▸(*b*).

The solutions of equation (6) are
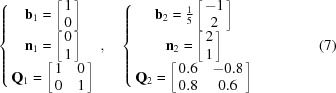
The first solution suggests [10](01) type dislocations or Σ1 boundaries on (01). Since **Q**_2_ is a 53.13° rotation (a 36.87° misorientation in the square lattice), the second solution suggests a Σ5 twin boundary on the (21) plane. The two solutions are illustrated in Fig. 4 ▸. The original crystal in structural state 1 is indicated by a dark-red square. Half of the material transforms to state 2. The boundaries between the two domains (in states 1 and 2) can be Σ1 or Σ5, as determined by the solution given in (7). In the case of Σ1, the two neighboring domains are in the same orientation but different structural states. In other words, the two domains separated by a Σ1 boundary in Fig. 4 ▸ are distinguished by different deformation states, *i.e.* **F**_1_ and **F**_2_, which can be identified in the displacement fields (as well as in the displacement gradient and deformation gradient). In particular, when one domain is within one atomic layer (*e.g.* the deformation gradient **F**_2_ is localized in one layer), it becomes a [10](01) type dislocation loop, and the symmetry-breaking process is associated with the motion of the dislocation (Fig. 4 ▸).

Similarly, considering the defects between the 2nd-NN vertices, we can apply equation (6) to other deformation gradient matrices. For example, [10](01) type dislocations or Σ1 boundaries can be generated between vertices 2 and 4. Boundaries with a 41.81° misorientation (non-special grain boundary) can be generated between vertices 2 and 5. Theoretically, we can systematically determine the possible types of defect generated between *m*th-NNs. However, those defects with a large *m* are usually difficult to generate by a real deformation. The choice of defects in a real crystal system could depend on the loading conditions, *e.g.* tensile/compressive/hydro­static stress, strain rate, temperature *etc.*, which dictate the competition between twinning and dislocation. However, we limit our analysis to pure crystallography in this work so that our approach generally applies to all kinds of crystal deformation, without any prior thermodynamic or kinetic knowledge of twinning and dislocations.

Note that the CDG is a group description beyond the reach of either point group or space group, as conventionally defined. The CDG takes the information from the LID into account. Geometrically, there are infinite types of LID for a given crystal, because of the translational symmetry. However, there are usually limited types of deformation mode that are energetically accessible from a physical point of view. For example, typical deformation modes in f.c.c. crystals are 1/2〈110〉{111} and 1/6〈112〉{111}, and typical deformation modes in body-centered cubic (b.c.c.) crystals are 1/2〈111〉{110} and 1/6〈111〉{112}. The construction of the CDG depends on the choice of deformation modes, which is a piece of information on the symmetry-breaking pathway during the deformation process. In the example of the square lattice in 2D, a special linear group can be obtained with the deformation mode of 〈10〉{01}. In the literature, special linear groups are also encountered in the definition of the lattice group (Parry, 1976 ▸; Ericksen, 1980 ▸; Pitteri, 1984 ▸; Fonseca, 1987 ▸; Bhattacharya *et al.*, 2004 ▸; Conti & Zanzotto, 2004 ▸; Gao, 2018 ▸). The so-called lattice group is introduced to provide a representation of the point group of a lattice, which is a particular way of describing crystal symmetry. Finite lattice groups are constructed through the subgroups of the special linear groups in 2D and 3D. Note that the lattice group (as well as the special linear group used in its definition) describes the symmetry of a lattice state, which is in contrast with the CDG. The CDG is a group capturing the symmetry-breaking process during crystal deformation, which relies on energetic information on the most favorable deformation mode. In the above 2D example, it is ‘coincidence’ that both the resulting groups are the SL_2_(Z) (special linear groups are widely investigated in mathematics since they have a large number of physical applications). However, CDGs are not necessarily special linear groups in general, which will be shown through the following example in 3D. In fact, the CDG of an f.c.c. crystal with a given deformation mode of 1/6〈112〉{111} has to be generated using the generating set presented below, which is not a special linear group (3D lattice group).

## 3. Analysis of deformation pathway and defects in f.c.c. crystals   

Here we consider a deformation mode in an f.c.c. crystal described by the following group generator:
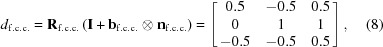
where **b**_f.c.c._ is a shear vector of 1/6[211] and **n**_f.c.c._ is the shear plane of 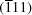. **R**_f.c.c._ is a rotation of 60° along the 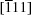 axis, which is included to make the deformation *d*_f.c.c._ produce an LID with the same orientation before and after deformation. It is clear that this deformation mode originates from the Shockley partial dislocation (or a Σ3 deformation twin) in f.c.c. crystals, and a full dislocation can also be captured since a full dislocation is the combination of two Shockley partials. As a result, the typical deformation modes observed in f.c.c. crystals are well described by the following generating set of the CDG:
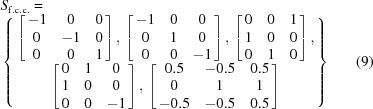
In the set of *S*_f.c.c._, the first four elements generate the proper rotational symmetry operations in an f.c.c. crystal, while the last one provides information on the LIDs. Note that the last element in the generating set does not belong to the special linear group SL_3_(Z), which requires all the entries in the matrix to be integers. As a result, it is clear that the CDG for an f.c.c. crystal is distinctly different from the lattice group of an f.c.c. crystal.

**Figure 5:**
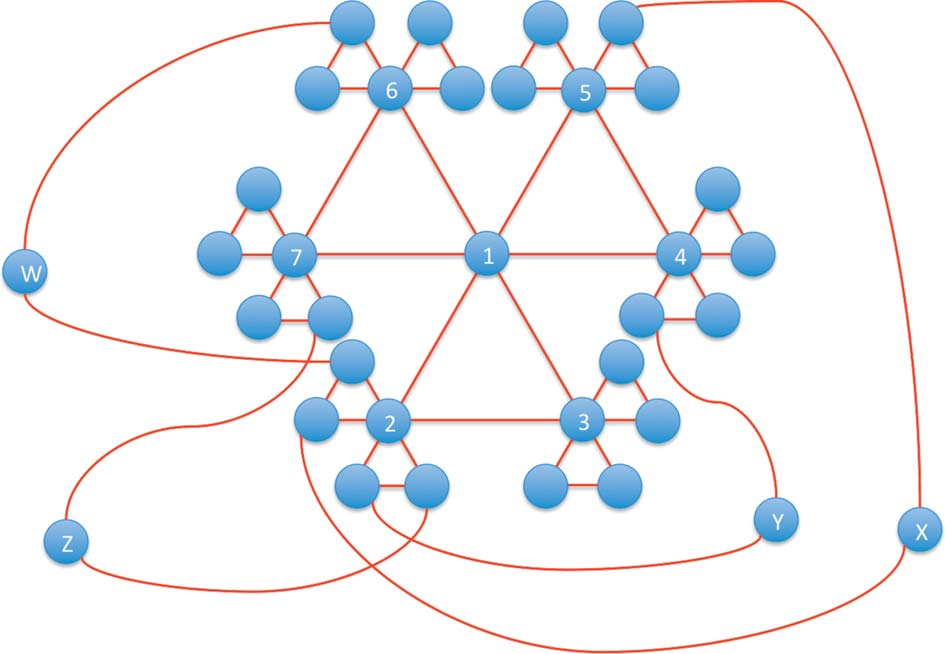
The deformation pathway graph for an f.c.c. crystal.

The DPG for an f.c.c. crystal is shown in Fig. 5 ▸. Because it is an infinite graph with a complex pathway network, only part of the graph is shown, so that we can focus on the 1st-, 2nd- and 3rd-NNs as well as the minimum circuit in this graph. For any given vertex (*e.g.* vertex 1), it has six 1st-NNs, 24 2nd-NNs and 84 3rd-NNs. Note that we can find 3-edge circuits and 6-edge circuits in Fig. 5 ▸. Furthermore, each vertex in this DPG is involved in three 3-edge circuits, and every two neighboring vertices are involved in one 3-edge circuit. Every two neighboring states are different by a deformation of 1/6〈112〉{111}. Each vertex is involved in twelve 6-edge circuits because there are four {111} planes and three 1/6〈112〉 on each plane in an f.c.c. crystal. For every two neighboring vertices, there exist four 6-edge circuits including both vertices (all four circuits including vertices 1 and 2 are shown in Fig. 5 ▸, through vertices W, X, Y and Z, respectively). For every three ‘non-triangular’ neighboring vertices (*e.g.* vertices 1, 2 and 4), there exists a unique 6-edge circuit (through Y) including all three vertices.

The DPG in Fig. 5 ▸ can be considered as an intrinsic characteristic of the deformation in f.c.c. crystals. As a result, the defect structures associated with the deformation can be determined systematically using a similar analysis to that of Fig. 3 ▸(*b*). By utilizing the kinematic compatibility condition [*i.e.* equation (6)], we can calculate the defects generated between 1st-NNs, 2nd-NNs and 3rd-NNs *etc*. The deformation gradient matrices for the structural states 1–4 (corresponding to vertices 1–4 in Fig. 5 ▸) are:
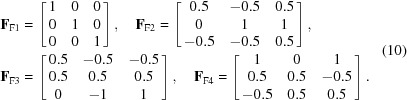



The defect structure between the 1st-NN structural states can be determined by utilizing equation (6) with **F**_F2_ and **F**_F1_, which leads to a Σ3 twin on either the 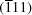 or (111) plane (in the **F**_F1_ index). If one domain is within one atomic layer, the Σ3 twin becomes a Shockley partial dislocation. Similarly, the defect structure between **F**_F3_ and **F**_F2_ is a Σ3 twin on either the (111) or 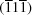 plane (in the **F**_F2_ index). In other words, the defects generated between 1st-NNs are a Σ3 twin or a Shockley partial dislocation. It is clear that the defects associated with 1st-NNs are directly related to the choice of the deformation mode *d*_f.c.c._, by comparing equations (6) and (8). In fact, an energetically favorable deformation mode is usually suggested by experimental observation of static defects, rather than direct observation of the atomic movements during a dynamic deformation process. However, the types of defect associated with 2nd- and higher-order-NNs are not easy to identify directly from *d*_f.c.c._, unless a DPG is constructed. Between the 2nd-NNs, *e.g.* **F**_F4_ and **F**_F2_, the defect structures are either a Σ1 boundary on the 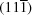 plane or a Σ11 twin on the 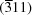 plane (in the **F**_F2_ index). If one domain is within one atomic layer, the Σ1 boundary becomes a full dislocation in an f.c.c. crystal, *i.e.* 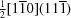. In a similar way, we can systematically determine the possible types of defect generated between *m*th-NNs, *e.g.* Σ5, Σ17*b* and Σ19*a* twins can be obtained between 3rd- and 4th-NNs, which could be related to the special grain boundaries observed after severe plastic deformation (Azzeddine *et al.*, 2015 ▸). Theoretically, the analysis of *m*th-NNs in CDGs and DPGs is analogous to the groupoid analysis of Σ3^*n*^ multiple twinning (Cayron, 2007 ▸). The former is subjected to the 1/6〈112〉{111} deformation between different structural states (in deformation space), while the latter is subjected to the 60° misorientation between different domains (in orientation space). Note that the relation between *m*th-NNs in a DPG is constrained by the compatibility condition [equation (6)] rather than the 60° misorientation (Gao *et al.*, 2018 ▸).

**Figure 6:**
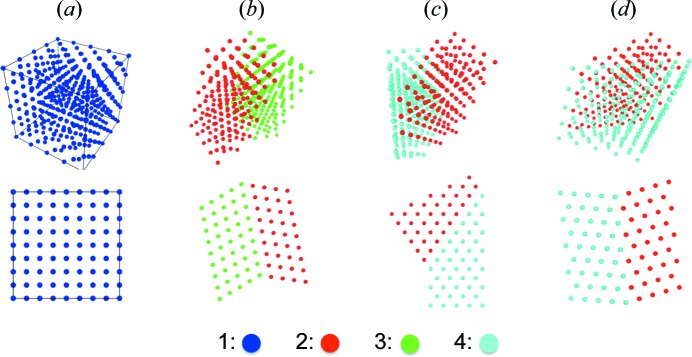
Atomic structures for twin boundaries generated by structural states **T**_1_–**T**_4_. (*a*) A single f.c.c. crystal at **T**_1_, (*b*) the Σ3 twin (**T**_2_/**T**_3_) on (111)_T2_, (*c*) the Σ1 twin (**T**_2_/**T**_4_) on 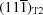 and (*d*) the Σ11 twin (**T**_2_/**T**_4_) on 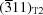.

Some of the above twin structures are illustrated in Fig. 6 ▸ using the *OVITO* visualization software (Stukowski, 2010 ▸). In Fig. 6 ▸(*a*), a single f.c.c. crystal structure with 64 (4 × 4 × 4) unit cells is constructed as the initial undeformed state at **F**_F1_. The initial single crystal is separated into two domains, which transform to two different structural states in Figs. 6 ▸(*b*)–6 ▸(*d*). The domains in different structural states are in different colors, and both the perspective view and the view along a specific crystallographic direction are shown for illustration purposes [〈100〉 for panel (*a*), 〈110〉 for panels (*b*)–(*d*)]. The Σ3 twin between **F**_F3_ and **F**_F2_ is shown in Fig. 6 ▸(*b*). The Σ1 and Σ11 twins between **F**_F4_ and **F**_F2_ are shown in Figs. 6 ▸(*c*) and 6 ▸(*d*), respectively. The mathematical details for the determination of the above twins are presented in Appendix *A* [App appa]. In previous experimental observations, the Σ3 twin has been reported as the dominant twin mode induced by conventional deformation in f.c.c. crystals (Merkle, 1991 ▸; Christian & Mahajan, 1995 ▸) because it originates from the 1st-NNs in the DPG. Other Σ twins have also been reported after severe plastic deformation (Azzeddine *et al.*, 2015 ▸). There has been no direct observation of the Σ11 deformation twin. However, the Σ11 twin has also been reported as a thermodynamically stable boundary in both experimental and theoretical studies (Merkle & Wolf, 1992 ▸; Mills *et al.*, 1992 ▸; Merkle, 1995 ▸; Kurtz *et al.*, 1999 ▸; Goukon *et al.*, 2000 ▸; Brown & Mishin, 2007 ▸; Mishin *et al.*, 2010 ▸). Meanwhile, both full and partial dislocations are generated during deformation, in agreement with our analysis of the 1st- and 2nd-NNs.

## 4. General procedure to determine the deformation pathway graph   

Based on the two examples shown above, we present here the general procedure to determine the CDG, DPG and defect structures for a given crystal with a given deformation mode (usually the energetically most favorable one).

(i) Ascertain the point group of the crystal. Determine a subgroup of the point group with all proper rotations (matrix representation) and determine the generators of this subgroup (*e.g.* *h*_1_).

(ii) For a given deformation, determine a lattice-invariant deformation matrix with a determinant of 1 (*e.g.* *d*_1_).

(iii) Using both the generators of the proper rotation group and the lattice-invariant deformation as a new generating set, we can obtain the CDG (*e.g.* *D*).

(iv) Draw the Cayley graph of the CDG (*e.g.* *G*) with the generating set determined in (iii).

(v) Simplify the Cayley graph to the DPG (*e.g.* *G^H^*) through a graph homomorphism.

(vi) Determine the defect structures between *m*th-NNs using geometric compatibility conditions.

The above method for constructing CDGs and DPGs can be applied to any given crystal system. In step (i), the generator of the point group of a given crystal can be found in the Bilbao Crystallographic Server (Aroyo, Kirov *et al.*, 2006 ▸; Aroyo, Perez-Mato *et al.*, 2006 ▸; Aroyo *et al.*, 2011 ▸). The choice of the energetically favorable deformation mode in step (ii) may not be unique in a given crystal, and is usually calculated based on experimental observations (see Appendix *B* [App appb] for more details). Steps (iii), (iv) and (v) can be performed following the standard methods of group theory and graph theory. The mathematical details for step (vi) can be found in classical phase-transformation crystallography theory (Wechsler *et al.*, 1953 ▸; Bowles & Mackenzie, 1954 ▸; Wayman, 1964 ▸; Bhattacharya, 2004 ▸).

## 5. Summary   

In this article, we have proposed a new theoretical framework to describe symmetry change and defect generation during plastic deformation of crystals. Using a combination of group theory and graph theory, we have established a crystal deformation group and a deformation pathway graph, which provide a systematic approach to predict the types of crystalline defect generated by the deformation. The new approach has been applied to f.c.c. crystals, and it has been demonstrated that a variety of types of twin boundary can be generated by deformation (besides conventional partial and full dislocations), *e.g.* Σ3, Σ11, Σ5, Σ17*b*, Σ19*a* *etc.*, which have been compared with experimental observations. The application of the deformation pathway graph could provide a new theoretical tool to guide defect engineering of crystalline materials.

## Appendix A Mathematics for the determination of defect structures in f.c.c. crystals

The equation to determine the defect between two structural states is

where **F**_F*j*_ and **F**_F*i*_ are the deformation gradient matrices of the structural states *j* and *i* [in Fig. 5 ▸ and equation (10)], respectively.

When *i* = 2 and *j* = 3, the solutions are

When *i* = 2 and *j* = 4, the solutions are

Note that all the above results are in the **F**_F1_ index. Fig. 6 ▸ is generated with the visualization software *OVITO*, using the above results.

## Appendix B Determination of crystal deformation groups in b.c.c. crystals

The choice of deformation mode is critical for the determination of a CDG, which is not unique for a given crystal. For a b.c.c. crystal, we consider two typical deformation modes, 1/6〈111〉{112} and 1/2〈111〉{110}, and the corresponding group generators are

There are three ways of generating a CDG for a b.c.c. crystal. If the 1/6〈111〉{112} deformation is dominant, the generating set of the CDG includes *d*_b.c.c.−1_,

It can be shown that the 1st-NN in its corresponding DPG generates a Σ3 twin using the compatibility condition.

If the 1/2〈111〉{110} deformation is dominant, the generating set includes *d*_b.c.c.−2_,

It can be shown that the 1st-NN in the corresponding DPG generates Σ1 and Σ11 twins using the compatibility condition. In the literature, both Σ3 and Σ11 twins are typically observed experimentally (Christian & Mahajan, 1995 ▸; Lai *et al.*, 2016 ▸).

If the two deformation modes are competing, the generating set includes both *d*_b.c.c.−1_ and *d*_b.c.c.−2_,

The DPG corresponding to the above group-generating set is complex, and will be determined in future work.
